# Effects of Confinement and Wheat Variety on the Performance of Two Aphid Species

**DOI:** 10.3390/insects16050477

**Published:** 2025-05-01

**Authors:** Maria Elisa D. A. Leandro, Joe M. Roberts, Ed T. Dickin, Tom W. Pope

**Affiliations:** Centre for Crop and Environmental Science, Agriculture and Environment Department, Harper Adams University, Newport, Shropshire TF10 8NB, UK; mdamascena@live.harper.ac.uk (M.E.D.A.L.); jroberts@harper-adams.ac.uk (J.M.R.); edicken@harper-adams.ac.uk (E.T.D.)

**Keywords:** *Rhopalosiphum padi*, *Sitobion avenae*, host plant resistance, clip cage, wheat varieties

## Abstract

Bird cherry-oat aphid (*Rhopalosiphum padi* L.; Hemiptera: Aphididae) and English grain aphid (*Sitobion avenae* Fabricius; Hemiptera: Aphididae) are economically important cereal crop pests and effective vectors of barley yellow dwarf virus (BYDV). While these aphid species have traditionally been managed with synthetic chemical insecticides, their use is increasingly difficult due to target organism resistance and potential non-target effects. Exploiting genetic diversity among cereal varieties offers a more sustainable control strategy. In this study, we evaluated how an experimental confinement method using clip cages to restrict an aphid to a single leaf versus free movement on the host plant affects the performance (growth and reproduction) of these two aphid species on various wheat varieties. Aphid performance was significantly influenced by both confinement and wheat variety. Notably, the two aphid species responded in opposite ways to confinement, with *S. avenae* growing quicker and producing a greater number of offspring under clip cage confinement compared to *R. padi*, which performed better when left free on the plant. This contrast is likely explained by species-specific feeding site preferences and sensitivity to the microenvironment created by the clip cages. We also found significant differences in aphid performance among host plant varieties, with both aphid species achieving their lowest growth rates on “Wolverine”, a modern BYDV-resistant wheat cultivar. Although none of the tested varieties were completely resistant to aphids, our results indicate that existing commercial cultivars may already carry partial resistance traits that can be leveraged in integrated pest management programs to help suppress aphid populations.

## Simple Summary

We compared how confining two common aphid species to a leaf section versus letting them move freely on different wheat plants (including old and modern varieties) affected their reproductive and growth success. We found that both the confinement method and the wheat variety influenced how well the aphids performed, likely due to species differences in feeding preferences and responses to microclimate. This highlights why choosing the right experimental method for the specific aphid being studied is important. The different wheat varieties also affected aphid performance, suggesting that useful resistance traits may exist in commercial wheat cultivars, even though none tested were fully resistant.

## 1. Introduction

Aphids are among the most economically important pests in cereal crops due to their role in vectoring plant viruses [1]. Bird cherry-oat aphid (*Rhopalosiphum padi* L.; Hemiptera: Aphididae) and English grain aphid (*Sitobion avenae* Fabricius; Hemiptera: Aphididae) vector barley yellow dwarf virus (BYDV) in cereal crops. Estimates suggest that BYDV may be responsible for yield losses of up to 84% in wheat and 64% in barley [2,3,4]. The impact on yield is mainly a result of reduced grain number [2]. Current BYDV management strategies include foliar pyrethroid insecticide applications and delayed sowing to avoid peak aphid migration events [5]. However, insecticide use is associated with negative impacts on non-target organisms and the evolution of resistance in the target organism to active ingredients that reduce the efficacy of key plant protection products [6,7]. Meanwhile, delayed sowing can result in reduced yields [8], although this effect can vary depending on wheat genetics and environmental conditions, particularly in regions without significant winter chilling requirements [9,10]. Where reduced yields are observed, this is due to low temperatures during crop vegetative growth and shortened duration of various phases of crop development [8].

Exploiting the genetic diversity found within different wheat varieties offers an alternative to current controls for managing both aphid pests and virus transmission [11,12]. Host plant genetics influence insect performance parameters, which may reduce aphid infestations through increased resistance [13]. This influence is multifaceted, stemming from variety-specific differences in: (i) physical traits like leaf toughness or trichome density that can impede movement or stylet penetration; and (ii) biochemical factors, including variations in essential amino acid profiles affecting nutritional quality, or the presence and concentration of defensive secondary metabolites (e.g., hydroxamic acids, phenolics) [14,15]. Aphid performance is typically measured using mean relative growth rate (MRGR) and intrinsic rate of increase (*r_m_*) to evaluate resistance or susceptibility across host plants, where reductions often indicate antibiosis or antixenosis effects [16,17,18,19]. Intrinsic rate of increase describes the rate at which a population changes size per unit of time by integrating both reproductive output and survival to provide a measure of population growth [20,21,22]. MRGR measures biomass gain relative to initial size over time, reflecting growth efficiency, which influences development speed and reproductive timing [23].

Confining individual aphids onto specific plant parts using clip-cages is a common practice in aphid performance studies to facilitate data collection and avoid losing individuals during experiments [11,24,25]. Although effective at containing aphids, clip cages have unintended consequences on their biology that may impact any conclusions drawn from performance studies [26]. For instance, as clip cages are designed to restrict aphid movement, they prevent an individual from choosing a feeding site. This is important as each aphid species may preferentially feed on specific plant parts [27]. Attaching and detaching clip cages during data collection may also damage plant tissue and upregulate defence responses in the host plant, indirectly affecting aphid development or limiting plant physiology in other ways [28,29]. Despite the widespread use of confinement methods, their potential to bias results, particularly when comparing species with different feeding behaviours, requires careful evaluation. To address this, the present study aimed to answer the following questions: (1) Does the experimental method (leaf confinement vs. whole-plant freedom) differentially affect the performance (MRGR and *r_m_*) of *R. padi* and *S. avenae*? (2) Does aphid performance vary significantly across selected modern and older wheat varieties, plus a standard barley control? (3) Is there an interaction between confinement method and cereal variety on aphid performance? Understanding these factors is crucial for accurately assessing host plant resistance and ensuring that screening methods reliably identify useful genetic traits for breeding aphid-resistant cereals.

## 2. Materials and Methods

### 2.1. Plant Material

Plants were grown under glasshouse conditions at Harper Adams University (52.777385, −2.427895) (mean temperature: 20 ± 5 °C/10 ± 5 °C day/night; 16 h:8 h light:dark photoperiod). Wheat (*Triticum aestivum* L.) and spring barley (*Hordeum vulgare* L.) seeds from each tested variety (Table 1) were sown 1 cm deep into 9 × 9 cm pots (Teku, Poeppelmann GmbH, Lohne, Germany) filled with peat-free John Innes No. 2 compost (Sylva Grow^®^, Melcourt, Tetbury, UK). Plants were grown in 60 × 60 × 60 cm fine nylon mesh (160 μm) cages (BugDorm-6E Insect Rearing Cage, Taichung, Taiwan) with a tray (58 × 58 cm) placed underneath the pots for watering until the plants had reached BBCH Growth Stage 12 (GS12) [30] before being used for experiments. The wheat varieties used in this study were selected to include a range of end uses and parental lineages (Table 1). Spring barley (var. Planet) was used to rear all aphid populations and included in experiments as a control to account for possible influence of previous generations’ feeding experience [31].

### 2.2. Aphid Populations and Age-Synchronised Cohorts

English grain aphid (*Sitobion avenae*) and bird cherry oat aphid (*Rhopalosiphum padi*) were established by collecting 10 mixed-age individuals of each species from cereal fields located at Harper Adams University and transferring them to potted spring barley (var. Planet) seedlings. Aphid infested barley plants were then placed in insect cages (47.5 × 47.5 × 47.5 cm) (Bugdorm-4 Insect Rearing Cage, Taichung, Taiwan) separated by species and housed in a plant growth room (Fitotron^®^ Weiss Technik, Loughborough, UK) maintained at 18 °C and 60% relative humidity under a 16:8 light:dark photoperiod. Population maintenance was carried out on a weekly basis by replacing heavily infested barley plants with clean plants. Each population of aphids was maintained in this way for over 10 generations before being used in experiments (i.e., approximately 10 to 12 weeks).

Age-synchronised aphid cohorts were produced prior to use in experiments to standardise the fitness of aphids at the start of each experiment. Aphid cohorts were established by transferring 20 adult aphids from the stock populations to clean barley plants (var. Planet) at BBCH GS12 in a new cage (47.5 × 47.5 × 47.5 cm) and left to larviposit for 24 h. Adult aphids were then removed, and only first instar nymphs were left to develop under the same conditions as stock populations until they had become adults.

### 2.3. Experimental Design

**Figure 1:**
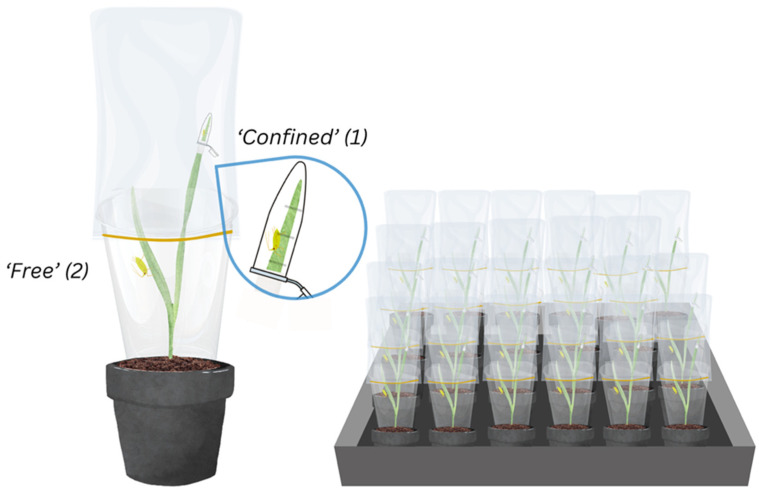
Host plant (wheat or barley) as in the aphid performance experiment (**left**) and experimental block consisting of 36 plants (**right**). Each aphid corresponds to one experimental unit in each of the methods tested simultaneously: “Free” (1) and “Confined” (2).

Aphid species were studied separately in consecutive experiments to avoid cross-contamination, and both experiments were completed in a plant growth room (Fitotron^®^ Weiss Technik, Loughborough, UK) maintained at 20 °C and 60% relative humidity under a 16:8 light:dark photoperiod. Two confinement methods (“confined” and “free”) were simultaneously tested on each experimental plant to facilitate direct comparison. Adults from age-synchronised cohorts were individually placed on each experimental plant, confined to a leaf section inside an open 1.5 mL microcentrifuge tube (Eppendorf AG, Hamburg, Germany) sealed with ¼ of a cotton pad (“confined”) or placed on whole plants covered with a clear plastic cylinder (13 cm in height, 7 cm in diameter at the top, and 5.5 cm in diameter at the bottom) mounted with a fine mesh organza bag at the top (18 × 13 cm) (“free”) (Figure 1). The leaf used for aphid confinement was randomly selected for each plant using a random number generator. Adults were left to larviposit for a 24 h period on experimental plants, and after 24 h, adult aphids and all but one first instar nymph were removed from each plant. Nymphs removed from these plants were weighed (XPR10/M Microbalance, Mettler Toledo, Columbus, OH, USA) in groups of ten to obtain a mean first-instar nymph weight. Each experimental nymph was carefully removed from the plant using a fine paintbrush (size 000) on day five, individually weighed, and then returned to the same plant and position from which it was taken. These aphids were monitored every day to track their development using exuviae. After reaching adulthood, each aphid was monitored every one to two days for a period equal to its development time, and the number of offspring was recorded and removed periodically. To evaluate aphid performance in the different host plants and confinement conditions, multiple biological parameters were measured and are described in Table 2, adapted from [31,32]. Five blocks with six replicates per treatment (variety) and two methods were carried out using a complete randomised block design for each aphid species.

### 2.4. Statistical Analysis

Data were first checked for normality (Shapiro–Wilk) and homoscedasticity (Levene’s). Because all datasets satisfied these assumptions, or were sufficiently close, linear mixed-effects models (LMMs) were fitted using the lme4 package [34], with “Replicate” as a random effect and “Variety” and “Method” as fixed effects. Separate models were fitted for each response variable (MRGR and *r_m_*), initially including the Variety × Method interaction. Likelihood ratio tests compared the full (interaction) model against the reduced (no-interaction) model, which was non-significant in all cases, and so final inferences were based on the reduced models. Post hoc pairwise comparisons were carried out using the emmeans package with Sidak adjustment [35]. All analyses were carried out using R (version 4.4.1).

## 3. Results

### 3.1. English Grain Aphid (Sitobion avenae)

#### 3.1.1. Mean Relative Growth Rate (MRGR)

**Figure 2:**
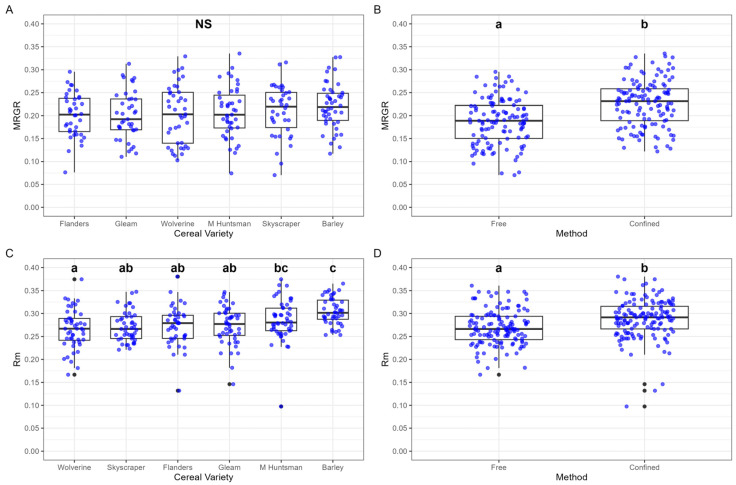
Effects of cereal variety and confinement method on the mean relative growth rate (MRGR) and intrinsic rate of increase (*r_m_*) in English grain aphid (*Sitobion avenae*). (**A**) MRGR across six cereal varieties, showing no significant difference; (**B**) MRGR under two confinement methods, with “Confined” resulting in significantly higher values than “Free”; (**C**) *r_m_* across six cereal varieties, with significant differences among varieties; (**D**) *r_m_* under two confinement methods, with “Confined” leading to significantly lower values compared to “Free.” Letters indicate significant differences using post hoc comparisons with Sidak correction (*p* < 0.05). Boxplots show median, interquartile range, and individual data points. Black dots illustrate data points 1.5 times the interquartile range beyond the box.

No differences were found in MRGR between cereal varieties (Figure 2A). Confinement method, however, had a significant effect, with a higher MRGR recorded for “confined” aphids MRGR (0.2267 ± 0.0042) compared to “free” aphids (0.1868 ± 0.0041) (Figure 2B).

#### 3.1.2. Intrinsic Rate of Increase (*r_m_*)

Significant differences were observed between cereal varieties. The highest *r_m_* (0.3048 ± 0.0038) was recorded for aphids feeding on barley, whilst the lowest *r_m_* (0.2658 ± 0.0057) was recorded on Wolverine (Figure 2C). Confinement also had a significant effect on *r_m_*, with higher values recorded for “confined” aphids (0.2879 ± 0.0031) compared to “free” aphids (0.2691 ± 0.0028).

### 3.2. Bird Cherry-Oat Aphid (Rhopalosiphum padi)

#### 3.2.1. Mean Relative Growth Rate (MRGR)

**Figure 3:**
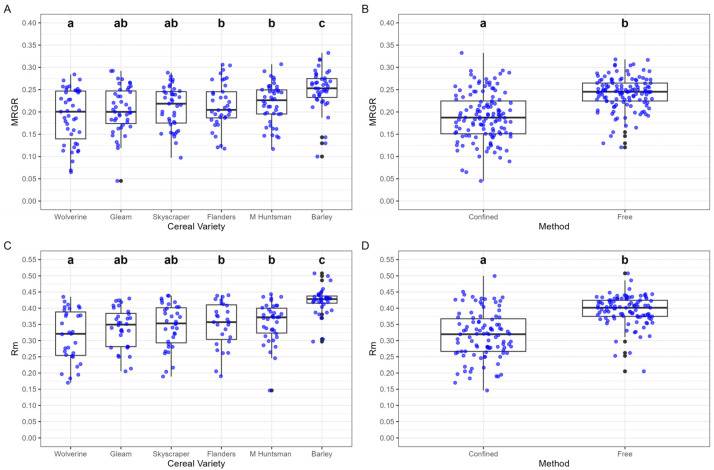
Effects of cereal variety and confinement method on the mean relative growth rate (MRGR) and intrinsic rate of increase (*r_m_*) of *R. padi*. (**A**) MRGR across six cereal varieties, showing significant differences among varieties; (**B**) MRGR under two confinement methods, with “Confined” showing significantly higher values than “Free”; (**C**) *r_m_* across six cereal varieties, with significant differences among varieties; (**D**) *r_m_* under two confinement methods, with “Confined” resulting in significantly lower values compared to “Free”. Letters indicate significant differences (*p* < 0.05), based on statistical analysis. Black dots illustrate data points 1.5 times the interquartile range beyond the box.

A significant effect of cereal variety was found on *R. padi* MRGR, but no significant interaction with confinement method was identified. The highest MRGR was recorded for aphids feeding on barley (0.2495 ± 0.0066), while the lowest was on Wolverine (0.1906 ± 0.0087) (Figure 3A). Wolverine-fed aphids exhibited significantly lower MRGR than those on the older wheat varieties Flanders (0.2142 ± 0.0073) and Maris Huntsman (0.2187 ± 0.0064) (Figure 3A). Confinement significantly reduced MRGR, with confined aphids exhibiting a lower growth rate (0.1892 ± 0.0045) compared to free aphids (0.2418 ± 0.0030), indicating that the restriction negatively impacted aphid growth (Figure 3B).

#### 3.2.2. Intrinsic Rate of Increase (*r_m_*)

There were significant differences in *R. padi r_m_* across cereal varieties and confinement methods. Among varieties, aphids feeding on barley exhibited the highest *r_m_* (0.4219 ± 0.0060), while those on Wolverine had the lowest (0.3146 ± 0.0115) (Figure 3C). Confinement had a significant negative effect on r_m_, with confined aphids having a lower intrinsic rate of increase (0.3171 ± 0.0061) compared to free aphids (0.3938 ± 0.0037), demonstrating a strong negative impact of restricted movement on aphid reproductive potential (Figure 3D).

## 4. Discussion

Results from this study demonstrate that the use of clip cages decreased *R. padi* performance in terms of both MRGR and *r_m_*, regardless of host plant or variety. As *R. padi* typically feeds at the base of the plant stem, particularly on younger plants [36], confinement to a leaf section is likely to compromise nutrient availability for this species. Similar findings have been reported in other aphid species, where confinement to non-preferred feeding sites alters growth and reproduction [37]. Other factors involving the microclimate formed in a confined space, such as increased humidity, may also play a role in aphid life history traits [37,38]. In contrast, the use of confinement increased the performance of *S. avenae*. An earlier study investigating the potential of a modified lightweight clip cage reported that the performance of this species was similar when confined to this clip cage or left free on the plant [39]. While it is not possible to directly compare the two studies, the contrasting confinement methods used further support the conclusion that the experimental method influences aphid performance recorded. However, the fact that these studies indicate a similar or increase in aphid performance when confined is likely to be, in part, due to the fact that this species typically feeds on leaves, and so the position of the confinement apparatus would have had a reduced effect on feeding behaviour.

Several studies have investigated resistance to both *S. avenae* and *R. padi* in wheat lines [11,39,40]. However, none of these studies have considered the impact that confinement may have on the aphid performance of both aphid species. This is likely to be most important where wheat collections, such as the Watkins and Gediflux collections, are screened [40], and the presence of partial plant resistance is masked by the impact of the experimental technique. The absence of partial plant resistance will have the impact of delaying and possibly preventing useful traits from being introgressed into elite breeding lines. The impact on aphid performance across cereal varieties highlights the role of plant genotype in shaping aphid population dynamics. Resistance mechanisms may include antixenosis, where aphids avoid specific varieties, and antibiosis, where host plant properties negatively impact aphid development and reproduction [41]. Previous work has reported significant variation in aphid performance when feeding on different lines within wheat collections [40]. The fact that we report reduced aphid performance on commercial varieties suggests that selective breeding, such as the inclusion of the Bdv2 gene to confer resistance to BYDV-PAV, may have unintentionally introduced traits that confer partial resistance to these pests [40]. This is important because recent work has shown that mixtures of wheat varieties have the potential to reduce the performance of *S. avenae* [42]. However, the combination of varieties appears to be important in achieving this effect, therefore, an understanding of aphid performance on and behavioural response to each wheat variety included in a mixture is likely to be an important factor in determining the success of this approach. In practice, a mixture strategy would need to account for both aphid species: a variety that strongly deters or slows *R. padi* might not have the same effect on *S. avenae*, and vice versa. Additionally, there are practical limitations to adopting a variety of mixtures on farms. Farmers often prefer monocultures for uniform crop management and marketability [43], so convincing them to plant mixtures could be challenging despite the potential pest control benefits. Nonetheless, the concept of tailored varietal mixes is a valuable addition to IPM, especially as legislative and environmental pressures increasingly limit chemical insecticide use. By combining cultivars that each contribute some level of aphid suppression or virus resistance, it may be possible to achieve more durable defense against pests like *R. padi* and *S. avenae* [32,44].

The higher performance of both aphid species on barley is consistent with findings from previous studies, where host plant suitability was linked to aphid fecundity and growth rates [24,45]. This may be attributed to nutritional factors, such as variations in plant secondary metabolites and amino acid profiles, which have been shown to influence aphid development [46]. The suitability of barley to both aphid species is likely to also reflect the rearing history of aphid populations on this host and is likely due to maternal effects. Maternal effects represent the impact of environmental variation in previous generations on phenotypic variations in the offspring generation. When reproducing parthenogenetically, asexual mother aphids develop telescoping generations (embryos within embryos) so that granddaughters are present inside the bodies of their grandmothers. As a result, aphids have strong maternal and transgenerational effects that can extend for three or more generations [47,48].

The aphid populations used in our study were reared on barley before the experiments, which could influence their performance through maternal or transgenerational effects [31,49]. In our case, the high performance observed on barley by both aphid species is likely influenced by this rearing history, as they were well-adapted to barley after being cultured on it. While we tested all varieties using aphids from the same rearing background, ensuring comparisons were still valid, this factor could limit the generality of the absolute performance values on each host. Future work could mitigate this by rearing aphids on the target host plants for one or two generations before measuring performance, or by using field-collected aphids that have not been long-term laboratory reared on a single host. Such steps would help in distinguishing true genetic host resistance effects from maternal conditioning effects. Additionally, experiments that explicitly examine how prior host (e.g., barley vs. wheat) affects aphid fitness on a subsequent host would be valuable to understand the extent of these maternal effects in aphid population dynamics. Our experiments were also conducted under no-choice conditions, meaning each aphid was initially placed on a given plant without the opportunity to choose a preferred host. This design is effective for assessing antibiosis, but it does not directly capture antixenosis [50].

Under real-world conditions, aphids are mobile and will actively select among available plant hosts. It is possible that some wheat varieties that appear susceptible in our no-choice test, because aphids can survive and reproduce on them when forced, might deter aphids from settling in a free-choice scenario. Conversely, a variety on which aphids had lower performance in our trials might still experience infestations if it lacks deterrent properties and aphids readily move onto it. To fully understand host resistance, future studies should incorporate choice tests or field trials where aphids can choose between different varieties. For instance, dual-choice assays or open-field observations could reveal if *R. padi* or *S. avenae* actively avoid certain wheat genotypes that were less favourable in our study [40,50]. Combining such preference data with performance data would allow researchers to distinguish between plants that are truly resistant in the field (both avoided and poor for growth) and those that are only low performance when feeding occurs but not avoided. This integrated understanding of antixenosis and antibiosis is crucial for breeding programs, as new cultivars should possess both attributes to reduce overall pest pressure. We also focused on two aphid species and a single genotype of each. As aphid populations are genetically diverse, different clones or biotypes may vary in their ability to cope with host defences or confinement stress. It has been demonstrated that aphid genotypes can differentially interact with host plants and experimental setups [26]. Our results for *R. padi* and *S. avenae* should, therefore, be interpreted as representative of the clones tested and not all genetic variants of these species. Future research should expand the diversity of aphid genotypes examined, including biotypes known to overcome certain plant resistances, to see if they respond similarly to confinement and host variety. Likewise, testing additional cereal varieties, including those from heritage collections or newly bred lines with potential resistance, would broaden the applicability of our findings.

This study underscores that the choice of experimental method significantly impacts aphid performance assessment in wheat in a species-dependent manner. Consequently, careful methodological validation, tailored to the target species, is crucial for reliably screening germplasm and avoiding misleading conclusions about host plant resistance, which could otherwise hinder progress towards less pesticide-reliant crop protection. While no variety exhibited complete resistance, the observed variation, including reduced aphid performance on the commercial variety Wolverine, indicates that valuable resistance traits may be present in existing cultivars. Harnessing these traits through breeding requires accurate assessment methods to efficiently develop improved varieties. Furthermore, deploying host plant resistance effectively within IPM strategies necessitates considering the specific aphid species present and the complexities of approaches like varietal mixtures versus broadly resistant single cultivars, highlighting the need for tailored IPM programs. Ultimately, integrating appropriate methodologies with an understanding of species-specific interactions is key to developing durable, genetically based aphid management solutions. Further research, particularly field validation and mechanistic studies, will be essential to translate these findings into practical, on-farm strategies.

## Figures and Tables

**Table 1:** Wheat varieties chosen for experiments.

Breeder	Wheat Variety	End Use Group ^1^	Parents
Desprez	Flanders	Old (1976–1983)	Champlein × FD 2816-348
Syngenta	Gleam	Hard group 4	Kielder × Hereford
Plant Breeding Institute	Maris Huntsman	Old (1972–1983)	[(CI 12633 × Cappellle Desprez × 5) × Hybrid 46] × Professeur Marchal
Limagrain	Skyscraper	Soft group 4	(Cassius × NAWW29) × KWS Santiago
RAGT	Wolverine	Hard group 4	(09TC2654 × Panorama) × Coronation

^1^ End use group based on UK classification: Hard group 4 = varieties suitable for bread making; Soft group 4 = varieties typically used for biscuits and cakes; Old = varieties registered pre-1985.

**Table 2:** Biological parameters measured to evaluate aphid performance on different wheat varieties.

Biological Parameter	Measurement
Development Time (DT)	Duration from birth to adult emergence + 0.5 d [31]
Weight Gain (Wg)	Wa − Wn ^1^ [31]
Mean Relative Growth Rate (MRGR)	(lnWa − lnWn)/DT [23,31]
Intrinsic Rate of Natural Increase (*r_m_*)	0.738 ln(fecundity)/DT [23,33]

^1^ Wa is the nymph weight 5 days after birth, Wn is the first-instar nymph weight (newborn within 24 h).

## Data Availability

The original data presented in the study are openly available in Figshare at https://doi.org/10.6084/m9.figshare.28890173.
